# Bis(isocyanato-κ*N*)bis­(1,10-phenanthroline-κ^2^ *N*,*N*′)cobalt(II)

**DOI:** 10.1107/S1600536810015758

**Published:** 2010-05-08

**Authors:** Dao-Peng Zhang, Nai-Chang Tian, Xiao-Mei Zhang

**Affiliations:** a School of Chemistry & Chemical Technology, Shandong University, Jinan 250100, People’s Republic of China

## Abstract

In the title complex, [Co(NCO)_2_(C_12_H_8_N_2_)_2_], the Co^II^ atom, lying on a twofold rotation axis, is coordinated in a distorted octa­hedral environment by four N atoms from two chelating phenanthroline ligands and two N atoms from two isocyanate ligands in *cis* positions.

## Related literature

For related structures, see: Cheng & Hu (2003 ▶); He *et al.* (2004 ▶); Yin (2007 ▶).
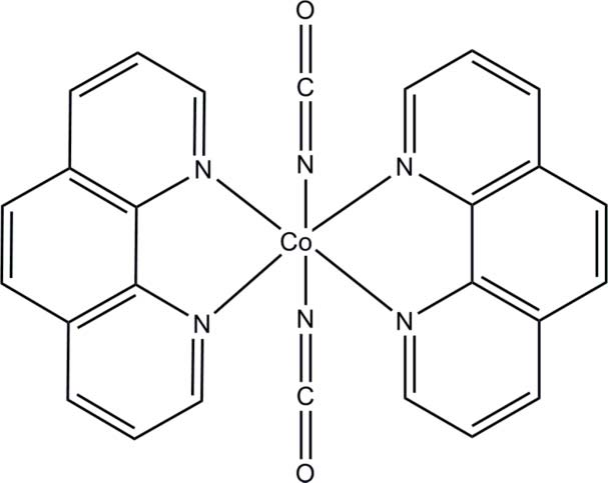


## Experimental

### 

#### Crystal data

[Co(CNO)_2_(C_12_H_8_N_2_)_2_]
*M* *_r_* = 503.38
Orthorhombic, 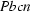

*a* = 13.2317 (8) Å
*b* = 9.7095 (6) Å
*c* = 16.7265 (10) Å
*V* = 2148.9 (2) Å^3^
*Z* = 4
Mo *K*α radiation
μ = 0.84 mm^−1^
*T* = 293 K
0.27 × 0.25 × 0.18 mm

#### Data collection

Bruker APEXII CCD diffractometer
Absorption correction: multi-scan (*SADABS*; Sheldrick, 1996 ▶) *T*_min_ = 0.805, *T*_max_ = 0.864
9949 measured reflections
1890 independent reflections
1553 reflections with *I* > 2σ(*I*)
*R*_int_ = 0.023

#### Refinement

*R*[*F* ^2^ > 2σ(*F* ^2^)] = 0.044
*wR*(*F* ^2^) = 0.144
*S* = 1.06
1890 reflections
159 parameters
H-atom parameters constrained
Δρ_max_ = 0.70 e Å^−3^
Δρ_min_ = −0.62 e Å^−3^

### 

Data collection: *APEX2* (Bruker, 2007 ▶); cell refinement: *SAINT-Plus* (Bruker, 2007 ▶); data reduction: *SAINT-Plus* (Bruker, 2007 ▶); program(s) used to solve structure: *SHELXS97* (Sheldrick, 2008 ▶); program(s) used to refine structure: *SHELXL97* (Sheldrick, 2008 ▶); molecular graphics: *SHELXTL* (Sheldrick, 2008 ▶); software used to prepare material for publication: *SHELXTL*.

## Supplementary Material

Crystal structure: contains datablocks I, global. DOI: 10.1107/S1600536810015758/hy2302sup1.cif

Structure factors: contains datablocks I. DOI: 10.1107/S1600536810015758/hy2302Isup2.hkl

Additional supplementary materials:  crystallographic information; 3D view; checkCIF report

## Figures and Tables

**Table 1:** Selected bond lengths (Å)

Co1—N1	2.168 (2)
Co1—N2	2.223 (3)
Co1—N3	2.058 (3)

**Fig. 1.**
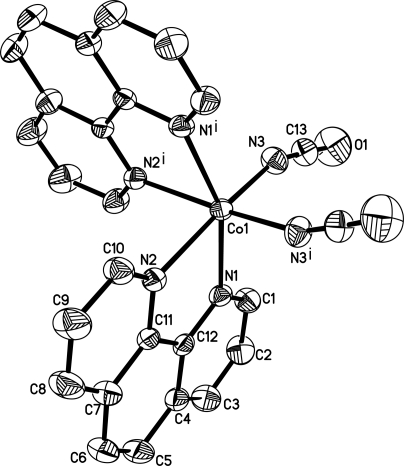
The molecular structure of the title compound. Displacement ellipsoids are drawn at the 30% probability level. H atoms are omitted for clarity. [Symmetry code: (i) 1-x, y, 3/2-z.]

## supplementary crystallographic information

### Comment

As has been known for a very long time, 1,10-phenanthroline (phen) is a good bidentate chelating ligand, and has been widely introduced into the transition metal complexes. Here, we present a new six-coordinated cobalt(II) complex based on phen.

The molecular structure of the title compound is shown in Fig. 1. The coordination geometry of the Co^II^ ion is distorted octahedral, in which four positions are occupied by four N atoms of two chelating phen ligands and the other two occupied by two N atoms of two isocyanate ligands at a *cis* position. The Co—N_phen_ and Co—N_isocyanate_ bond lengths are 2.168 (2), 2.223 (3) and 2.058 (3) Å (Table 1), respectively, which are all comparable to those found in other bis(phen)cobalt(II) complexes (Cheng & Hu, 2003; He *et al.*, 2004; Yin, 2007).

### Experimental

To a solution of 1,10-phenanthroline monohydrate (39.6 mg, 0.2 mmol) dissolved in methanol (15 ml) was added Co(ClO_4_)_2_.6H_2_O (36.6 mg, 0.1 mmol). The mixture was stirred for 5 min before NaNCO (13 mg, 0.2 mmol) was added. After the stirring process was continued for an additional 5 min, the mixture was filtered, and the filtrate was allowed to slow evaporate to afford orange-yellow crystals suitable for X-ray diffraction with a yield about 55%.

### Refinement

H atoms were positioned geometrically and refined as riding atoms, with C—H = 0.93 Å and with *U*_iso_(H) = 1.2*U*_eq_(C).

### Crystal data

**Table:**

[Co(CNO)_2_(C_12_H_8_N_2_)_2_]	*F*(000) = 1028
*M**_r_* = 503.38	*D*_x_ = 1.556 Mg m^−^^3^
Orthorhombic, *P**b**c**n*	Mo *K*α radiation, λ = 0.71073 Å
Hall symbol: -P 2n 2ab	Cell parameters from 1231 reflections
*a* = 13.2317 (8) Å	θ = 2.6–27.0°
*b* = 9.7095 (6) Å	µ = 0.84 mm^−^^1^
*c* = 16.7265 (10) Å	*T* = 293 K
*V* = 2148.9 (2) Å^3^	Block, orange-yellow
*Z* = 4	0.27 × 0.25 × 0.18 mm

### Data collection

**Table:**

Bruker APEXII CCD diffractometer	1890 independent reflections
Radiation source: fine-focus sealed tube	1553 reflections with *I* > 2σ(*I*)
graphite	*R*_int_ = 0.023
φ and ω scans	θ_max_ = 25.0°, θ_min_ = 2.4°
Absorption correction: multi-scan (*SADABS*; Sheldrick, 1996)	*h* = −15→15
*T*_min_ = 0.805, *T*_max_ = 0.864	*k* = −7→11
9949 measured reflections	*l* = −19→19

### Refinement

**Table:**

Refinement on *F*^2^	Primary atom site location: structure-invariant direct methods
Least-squares matrix: full	Secondary atom site location: difference Fourier map
*R*[*F*^2^ > 2σ(*F*^2^)] = 0.044	Hydrogen site location: inferred from neighbouring sites
*wR*(*F*^2^) = 0.144	H-atom parameters constrained
*S* = 1.06	*w* = 1/[σ^2^(*F*_o_^2^) + (0.0851*P*)^2^ + 1.8714*P*] where *P* = (*F*_o_^2^ + 2*F*_c_^2^)/3
1890 reflections	(Δ/σ)_max_ = 0.001
159 parameters	Δρ_max_ = 0.70 e Å^−^^3^
0 restraints	Δρ_min_ = −0.62 e Å^−^^3^

### Fractional atomic coordinates and isotropic or equivalent isotropic displacement parameters (Å^2^)

**Table:**

	*x*	*y*	*z*	*U*_iso_*/*U*_eq_	
Co1	0.5000	0.18820 (5)	0.7500	0.0357 (2)	
O1	0.3547 (3)	0.4750 (4)	0.9117 (2)	0.1113 (12)	
N1	0.34514 (17)	0.1463 (3)	0.71529 (15)	0.0407 (6)	
N2	0.51307 (17)	0.0223 (3)	0.65894 (15)	0.0402 (6)	
N3	0.4590 (2)	0.3270 (3)	0.83709 (18)	0.0551 (7)	
C1	0.2633 (3)	0.2026 (4)	0.7466 (2)	0.0519 (9)	
H1	0.2713	0.2725	0.7841	0.062*	
C2	0.1656 (3)	0.1624 (4)	0.7262 (3)	0.0636 (10)	
H2	0.1099	0.2033	0.7503	0.076*	
C3	0.1531 (3)	0.0611 (4)	0.6696 (3)	0.0694 (11)	
H3	0.0886	0.0323	0.6553	0.083*	
C4	0.2379 (2)	0.0016 (3)	0.6335 (2)	0.0507 (8)	
C5	0.2324 (3)	−0.1020 (4)	0.5729 (3)	0.0721 (11)	
H5	0.1695	−0.1280	0.5532	0.086*	
C6	0.3168 (3)	−0.1634 (4)	0.5432 (2)	0.0705 (11)	
H6	0.3108	−0.2326	0.5050	0.085*	
C7	0.4141 (3)	−0.1225 (4)	0.5701 (2)	0.0581 (9)	
C8	0.5032 (3)	−0.1852 (5)	0.5447 (3)	0.0720 (14)	
H8	0.5003	−0.2574	0.5082	0.086*	
C9	0.5947 (3)	−0.1417 (5)	0.5727 (2)	0.0759 (12)	
H9	0.6545	−0.1807	0.5542	0.091*	
C10	0.5961 (3)	−0.0358 (4)	0.6307 (2)	0.0548 (8)	
H10	0.6583	−0.0057	0.6499	0.066*	
C11	0.4227 (2)	−0.0189 (3)	0.62939 (17)	0.0402 (7)	
C12	0.3333 (2)	0.0450 (3)	0.65996 (17)	0.0410 (7)	
C13	0.4114 (3)	0.4017 (4)	0.8731 (2)	0.0548 (8)	

### Atomic displacement parameters (Å^2^)

**Table:**

	*U*^11^	*U*^22^	*U*^33^	*U*^12^	*U*^13^	*U*^23^
Co1	0.0293 (4)	0.0381 (4)	0.0396 (4)	0.000	−0.0021 (2)	0.000
O1	0.112 (3)	0.117 (3)	0.105 (3)	0.031 (2)	0.026 (2)	−0.026 (2)
N1	0.0337 (13)	0.0439 (13)	0.0446 (14)	−0.0007 (11)	−0.0062 (11)	0.0051 (11)
N2	0.0395 (13)	0.0402 (14)	0.0409 (13)	0.0017 (10)	−0.0029 (10)	−0.0013 (11)
N3	0.0524 (17)	0.0535 (16)	0.0593 (17)	0.0016 (13)	0.0047 (15)	−0.0093 (14)
C1	0.0381 (19)	0.055 (2)	0.062 (2)	0.0014 (15)	−0.0010 (13)	−0.0053 (15)
C2	0.0308 (17)	0.068 (2)	0.092 (3)	−0.0021 (16)	0.0034 (18)	0.002 (2)
C3	0.0423 (19)	0.071 (2)	0.095 (3)	−0.0157 (18)	−0.0190 (19)	0.004 (2)
C4	0.0464 (18)	0.0461 (17)	0.0598 (19)	−0.0053 (14)	−0.0155 (15)	0.0057 (15)
C5	0.065 (2)	0.071 (2)	0.080 (3)	−0.020 (2)	−0.032 (2)	−0.004 (2)
C6	0.080 (3)	0.064 (2)	0.067 (2)	−0.015 (2)	−0.021 (2)	−0.0126 (19)
C7	0.070 (2)	0.058 (2)	0.0467 (18)	−0.0031 (18)	−0.0113 (17)	−0.0070 (16)
C8	0.073 (3)	0.081 (3)	0.062 (3)	0.0094 (19)	−0.0093 (18)	−0.032 (2)
C9	0.073 (3)	0.095 (3)	0.060 (2)	0.021 (2)	0.000 (2)	−0.030 (2)
C10	0.0512 (19)	0.060 (2)	0.0529 (19)	0.0070 (16)	−0.0040 (15)	−0.0139 (16)
C11	0.0435 (16)	0.0374 (15)	0.0398 (15)	−0.0038 (12)	−0.0097 (13)	0.0058 (12)
C12	0.0440 (16)	0.0365 (14)	0.0426 (15)	−0.0042 (12)	−0.0106 (13)	0.0112 (12)
C13	0.055 (2)	0.0567 (19)	0.0525 (19)	0.0017 (17)	0.0092 (16)	−0.0045 (16)

### Geometric parameters (Å, °)

**Table:**

Co1—N1	2.168 (2)	C4—C12	1.403 (4)
Co1—N2	2.223 (3)	C4—C5	1.430 (5)
Co1—N3	2.058 (3)	C5—C6	1.360 (6)
O1—C13	1.220 (4)	C5—H5	0.9300
N1—C1	1.321 (5)	C6—C7	1.420 (5)
N1—C12	1.359 (4)	C6—H6	0.9300
N2—C10	1.322 (4)	C7—C8	1.394 (5)
N2—C11	1.354 (4)	C7—C11	1.418 (5)
N3—C13	1.133 (4)	C8—C9	1.365 (6)
C1—C2	1.393 (5)	C8—H8	0.9300
C1—H1	0.9300	C9—C10	1.414 (5)
C2—C3	1.374 (6)	C9—H9	0.9300
C2—H2	0.9300	C10—H10	0.9300
C3—C4	1.399 (5)	C11—C12	1.430 (4)
C3—H3	0.9300		
			
N3^i^—Co1—N3	98.13 (17)	C4—C3—H3	120.1
N3^i^—Co1—N1	100.52 (11)	C3—C4—C12	117.5 (3)
N3—Co1—N1	93.64 (11)	C3—C4—C5	123.8 (3)
N3^i^—Co1—N1^i^	93.64 (11)	C12—C4—C5	118.7 (3)
N3—Co1—N1^i^	100.52 (11)	C6—C5—C4	121.7 (3)
N1—Co1—N1^i^	158.36 (13)	C6—C5—H5	119.2
N3^i^—Co1—N2	88.23 (11)	C4—C5—H5	119.2
N3—Co1—N2	168.55 (10)	C5—C6—C7	120.4 (4)
N1—Co1—N2	75.76 (9)	C5—C6—H6	119.8
N1^i^—Co1—N2	88.51 (9)	C7—C6—H6	119.8
N3^i^—Co1—N2^i^	168.55 (10)	C8—C7—C11	117.1 (3)
N3—Co1—N2^i^	88.23 (11)	C8—C7—C6	123.3 (3)
N1—Co1—N2^i^	88.51 (9)	C11—C7—C6	119.5 (4)
N1^i^—Co1—N2^i^	75.76 (9)	C9—C8—C7	120.7 (4)
N2—Co1—N2^i^	87.17 (13)	C9—C8—H8	119.6
C1—N1—C12	118.3 (3)	C7—C8—H8	119.6
C1—N1—Co1	126.2 (2)	C8—C9—C10	118.2 (4)
C12—N1—Co1	115.26 (19)	C8—C9—H9	120.9
C10—N2—C11	118.5 (3)	C10—C9—H9	120.9
C10—N2—Co1	128.2 (2)	N2—C10—C9	123.0 (3)
C11—N2—Co1	113.28 (19)	N2—C10—H10	118.5
C13—N3—Co1	160.3 (3)	C9—C10—H10	118.5
N1—C1—C2	123.2 (3)	N2—C11—C7	122.4 (3)
N1—C1—H1	118.4	N2—C11—C12	118.1 (3)
C2—C1—H1	118.4	C7—C11—C12	119.5 (3)
C3—C2—C1	118.7 (4)	N1—C12—C4	122.4 (3)
C3—C2—H2	120.6	N1—C12—C11	117.5 (2)
C1—C2—H2	120.6	C4—C12—C11	120.1 (3)
C2—C3—C4	119.8 (3)	N3—C13—O1	175.3 (4)
C2—C3—H3	120.1		

Symmetry codes: (i) −*x*+1, *y*, −*z*+3/2.
